# Unconscious Priming of Task-Switching Generalizes to an Untrained Task

**DOI:** 10.1371/journal.pone.0088416

**Published:** 2014-02-06

**Authors:** Tom Manly, Jessica E. Fish, Sarah Griffiths, Meike Molenveld, Fanzhi A. Zhou, Greg J. Davis

**Affiliations:** 1 Medical Research Council Cognition and Brain Sciences Unit, Cambridge, United Kingdom; 2 King's College London, Institute of Psychiatry, Department of Psychology, London, United Kingdom; 3 University of Cambridge Department of Experimental Psychology, Cambridge, United Kingdom; Goldsmiths, University of London, United Kingdom

## Abstract

Evidence suggests that subliminal stimuli can influence ostensibly volitional, executive processes but it is unclear whether this is highly task-specific. To address this we used a set-switching task. Volunteers saw a word pair and reported either if both words had the same number of syllables or if both were concrete. Task selection was random and instructed by a hexagon/triangle preceding the words. A subliminally-presented square or diamond reliably preceded each of these consciously perceived instruction-shapes. Significant congruency effects were observed in a subsequent Test Phase in which primes no longer reliably predicted the task (and in which high/low tones now served as conscious instructions). The Generalization Phase required novel phonological (rhyme) or semantic (category) judgments. Remarkably, unconscious priming congruency effects carried over in those participants who had shown priming in the Test Phase, the degree correlating across the two conditions. In a final phase of the study, participants were asked to discriminate between the two originally presented prime shapes. Those participants whose discriminations were more accurate showed reduced priming relative to participants with less accurate discriminations. The results suggest that, rather than being highly task specific, priming can operate at the level of a generalizable process and that greater awareness of primes may lessen their impact on behavior.

## Introduction

Events that occur outside awareness can nevertheless influence perceptual, semantic and motor functions. Words or pictures presented at durations too brief to allow identification can, for example, speed identification of a subsequent related word or image. There is an extensive literature on such perceptual, semantic and motor priming effects [1], [2], [3]. Only recently have studies begun to investigate whether ostensibly volitional executive processes can also be primed. Lau and Passingham ([4] see also Mattler [5]) asked healthy volunteers to switch between two sorts of tasks (“mental sets”). Such task-switching is invariably associated with a time-cost compared with the consistent application of a single-task, even when the switch can be anticipated [6]. In Lau and Passingham's paradigm participants were presented with a word and asked either to judge whether it had two syllables or whether it was concrete. The random ordering of trials meant that participants could not anticipate the relevant rule until an instruction (a diamond or square) appeared just before the word. However, for an extremely brief period (33 msec.) prior to this conscious instruction, a subliminal diamond or square prime appeared. Although unable to see or identify it, responses were significantly faster following a congruent prime-instruction pair than an incongruent pair. Furthermore, functional magnetic resonance imaging suggested that, on incongruent trials, brain regions associated with the primed but irrelevant task (e.g. areas linked to phonological analysis during a concrete judgment trial) were more active in comparison with a congruent condition. Acknowledging the difficulty in assessing the absolute visibility of an ‘unconscious’ stimuli, Lau and Passingham included a third condition in which the prime was rendered more visible and, remarkably, reported a dissociation in which the more visible prime had no detectable effect on switching performance.

Weibel et al. [7] sought to address concerns that the visibility of the unconscious primes in Lau and Passingham's study may have been influenced by the presence of these identical visible primes. Accordingly they administered a phonological/semantic word judgment set-switching paradigm in which participants were never consciously exposed to primes. In the design a consciously perceived letter indicating the task to perform was presented 156 msec. before the word. Prior to this (36 msec. Experiment 1, 84 msec. Experiment 2) the same letter had been presented for a very brief (12 msec.) period followed by a mask. A condition was also included in which the task was only to discriminate the conscious instruction in order to examine whether the apparent priming of task-set may in fact be perceptual priming of the instruction. At short SOAs the primes indeed sped recognition of the subsequent (if identical) conscious instruction letter but did not produce facilitation/interference with reaction time on the word judgment task. In contrast, at the longer SOA (i.e. closer to that used by Lau and Passingham) perceptual priming of the instruction letter and ostensible task-set priming occurred.

This result highlights a difficulty in fully interpreting the pioneering behavioral work of Mattler [5] and Lau and Passingham [4]; whether, due to the use of identical stimuli for prime and conscious instructions, the effects reflect speeding identification of the conscious instruction (i.e. perceptual priming) or unconscious priming of task set. Seeking to address this issue, Reuss et al. [8] employed a switching paradigm in which, on key trials, there was no conscious instruction. In the task participants had either to judge the magnitude of a digit (><5) or its odd/even status. Half of the trials by alternation had a clearly visible instruction, one of two letters arbitrarily linked with the tasks. In the other trials the same letter primes were rendered invisible or less visible by brevity (30 msec.) and masking. Participants were asked to look out for instruction letters and, if one was seen, perform the indicated task. If no instruction was seen, they should choose which of the two tasks to perform, selecting each with approximately equal frequency. In trials with a visible instruction, participants were accurate (93%) and showed the expected switch-cost in reaction times and accuracy when the current trial required a different task to that previously chosen. Despite the apparent free-choice on masked instruction trials, these deviated moderately but significantly from chance in the direction of the hidden instruction (53.9% congruent). Although an issue in this study was that discrimination of the hidden instruction on post-test was also above chance (53.3%), the magnitude of priming effect was not correlated with detection rates. As the authors point out, the possibility of apparent task-set priming resulting from perceptual priming of a subsequent consciously perceived instruction is ruled out by the absence of a seen instruction in the ‘free choice’ trials. Notably, however, in this paradigm as with that of Lau and Passingham[4], the unconscious prime was identical to the instruction consciously linked with each task.

Zhou and Davis [9] circumvented problems related to perceptual priming of instructions and the possibility that the unconscious prime might trigger the learned association between a consciously perceived instruction and task-set. They (Experiments 2A and 2B) adapted Lau and Passingham's switching paradigm and introduced a learning phase in which two subliminal shapes reliably preceded each of two (different) consciously perceived instruction-shapes but were never themselves made conscious. Participants were not told about the primes nor were they able to identify them on post-test when they now knew which shapes to look for and were attending to their occurrence. Nevertheless, in a test phase where prime-task pairings were random, there was a significant congruency effect based on the training phase. The possibility of this resulting from perceptual priming of the conscious instruction was abolished both by the prime and instruction shapes differing during learning, and the conscious instruction changing from shapes to tones in the test phase. An interesting additional finding from Zhou and Davis' studies was that deliberately directing participants' attention away from the location of the unconscious prime did not erode the priming task-set congruency effect, indeed, in some experiments this appeared to strengthen it.

In summary, whilst there is some skepticism about unconscious priming of task set and whether effects may be more parsimoniously related to prime visibility or perceptual priming etc., careful analyses in different laboratories in recent years have increased confidence that such influences can be detected. An issue is that level at which such priming may operate; whether, for example, it is highly specific to the particular tasks/stimulus set etc. used or could generalize to a novel task that required related cognitive processes. To address this issue we adapted Zhou and Davis' [9] paradigm. In each trial of our Training Phase two words were simultaneously presented on the screen and participants were asked to judge either whether they had the same number of syllables or whether both represented concrete concepts. This was disambiguated by one of two consciously perceived instruction shapes (hexagon/triangle) that preceded the words. Earlier in each trial one of two subliminal prime shapes (square/diamond) was presented, each reliably paired over the block with one of the subsequent instruction shapes. In the Test Phase, during which each prime preceded the syllable and concrete task with equal frequency, we examined whether congruency effects related to the Training Phase were detectable. The use of word pairs allowed us to develop a further Generalization Phase. Here, word-pairs were again presented but now participants were asked to judge either whether they rhymed or whether they were from the same common semantic category (e.g. both fruits, furniture etc.). If congruency effects persisted it would suggest primes were operating at a more general level of process, biasing participants towards phonological analysis rather than just syllable enumeration or towards semantic analysis rather than just concrete/abstract decisions.

## Method

### Participants

The study was given ethical approval by the Cambridge Psychology Research Ethics Committee (ref: TM99). Thirty-three participants (all over 18 years old, mean age 26.88, SD 9.10, 22 women) were recruited from the Medical Research Council Cognition and Brain Sciences Unit Volunteer Panel and gave informed written consent for their participation. Participants were all native English speakers with no history of neurological disorder and with normal or corrected to normal vision and hearing.

### Experimental task

#### Training Phase

**Figure 1:**
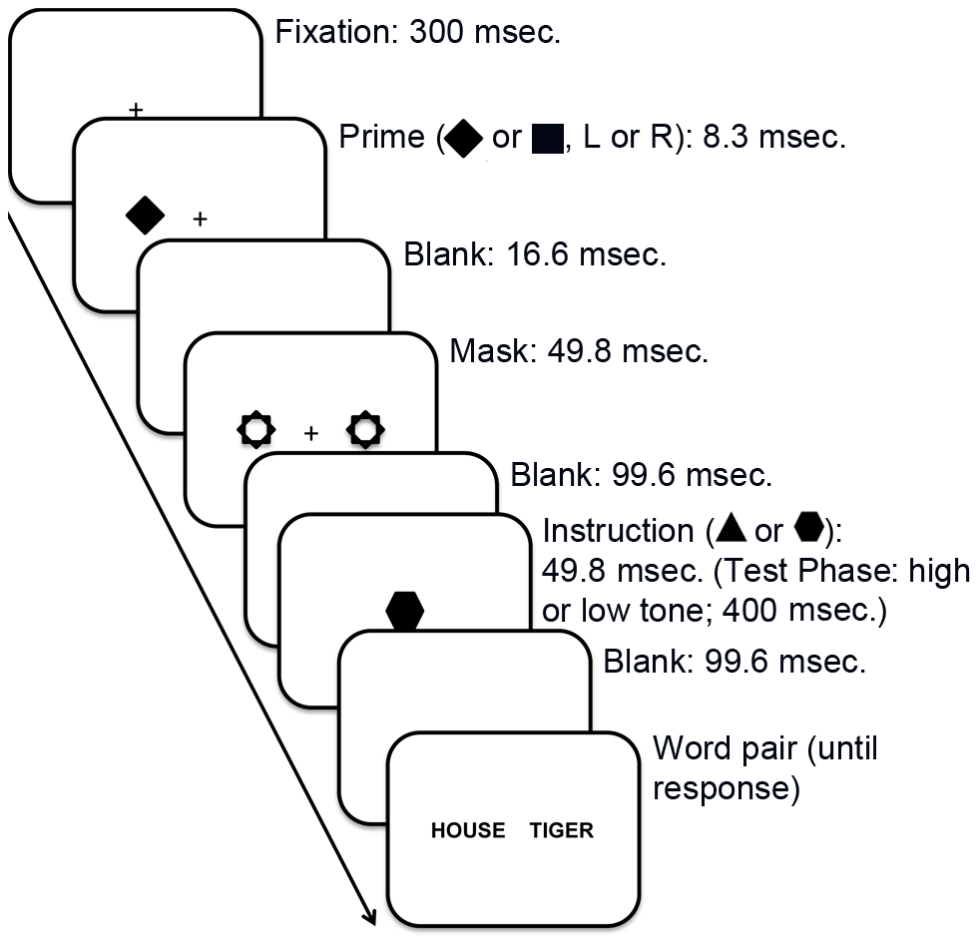
Sequence of events in each trial of the Training, Test and Generalization Phases. A central fixation cross was presented for 300 msec. followed by a diamond or square prime to the left or right of fixation for 8.3 msec. Following a blank screen of 16.6 msec duration two masks were presented in both of the potential prime locations and remained on screen for 49.8 msec followed by a blank screen of 99.6 msec. The conscious instruction was then presesented. In the Training Phase this consisted of a central black triangle or hexagon for 49.8 msec. In the Test and Generalization Phases this was replaced by a high or low tone of 400 msec. duration. Following a further blank screen of 99.6 msec., the word pair was presented which remained on screen until a response was made.

Trials in the Training Phase (see figure 1) began with a black central fixation cross presented against a uniform white background of a 300×400 mm cathode ray tube computer monitor (refresh rate 120 Hz). After 300 msec., a black prime (square or diamond 16×16 mm) appeared for 8.3 msec. to the left or right side of the fixation cross (which remained on-screen). Both prime and cross then disappeared for 16.6 msec., after which two visual masks, formed by the superimposed outlines of the square and diamond, were presented simultaneously in both possible prime locations for 49.8 msec. The duration and masking were designed to render the primes subliminal. Following another blank screen for 99.6 msec., a central black hexagon or triangle (16×16 mm) was then presented for 49.8 msec., followed by a blank screen for 99.6 msec. This unmasked shape was the consciously perceived instruction cue indicating which of the two tasks participants were to perform on the subsequently presented word pair (hexagon-syllables, triangle-concrete). Words were presented 7.5 cm apart in the center of the screen in black capital letters (4 mm in height). The words remained on the screen until the participant's response was registered. The same, labeled response keys were used for both tasks (QWERTY N  =  same, M  =  different).

Training Phase pairings were always square subliminal prime followed by conscious triangle instruction indicating concrete/abstract judgment or diamond subliminal prime followed by conscious hexagon instruction indicating syllable number judgment. The 96 training trials were cross-balanced in terms of task and correct same/different response. Trials in each task were mixed in terms of whether the response would be congruent in the other task (e.g. concrete-concrete pairs could have the same or different number of syllables).

#### Test Phase

The 64 trials in the Test Phase were identical to those of the Training Phase with the following exceptions. The prime-task pairings were now 50% congruent with those of the Training Phase and 50% incongruent. To abolish any purely perceptual priming, as with Zhou and Davis [9], the conscious instruction for the concrete task was now a high (437.32 Hz) tone of 400 msec. duration and the instruction for the syllables task was now a low tone (210.68 Hz, 400 msec.). As with the Training Phase, the proportions of concrete/syllable trials and same/different trials were cross-balanced.

#### Generalization Phase

The 64 trials of the Generalization Phase were identical to those of the Test Phase with one exception. The low tone instructed participants to judge whether the words rhymed (“same”) or not (“different”). A high tone instructed them to report whether or both words were from the same semantic category. The categories (furniture, animals, clothing, fruits, emotions and concepts relating to justice) were selected so as to include concrete and abstract words. Participants were told the categories in the Generalization Phase instructions.

#### Prime identification test

Finally participants were shown pictures of the diamond and square primes and then asked to complete a 64-item forced-choice diamond/square identification task in which the primes were presented in random order. Presentation was as in the previous phases but with no subsequent conscious instruction or word pair.

### Generation of word-pair stimuli

A word list was generated using the MRC Psycholinguistic database (http://www.psy.uwa.edu.au/MRCDataBase/uwa_mrc.htm), with the search criteria being: 4-12 letters, 1-3 syllables, Brown verbal frequency rating of 1-5000. Archaic and abbreviated words were excluded. For the syllable judgment trials, 160 words were selected from the one, two or three syllables lists and then randomly paired such that half matched in this respect. Words with relatively high and low concreteness values (>550 or <350) were used for the syllable and concrete task pairs.

For the rhyming task the first word was also selected where possible from the high and low concreteness list. Rhymes in which both phonology and orthography matched (SORROW BORROW) and where only phonology matched (PLAYS RAISE) were used. The constraints of generating rhyming word pairs meant it was no longer possible to balance the presentation of items between the two task types of the previous blocks; relatively few words relating to the semantic categories appeared in the rhyme task pairs and there were no trials in which both semantic category words rhymed. Whilst this would arguably reduce response conflict, the key question here concerned prime-task congruency.

### Procedure

After providing informed consent, participants were tested individually in a quiet slightly darkened room. They sat at a comfortable viewing distance from the monitor. Participants were given initial instructions about the overt structure of each trial, the two tasks and examples of concrete and abstract words. Participants were not told about the primes at this stage. They then completed the Training Phase. Before the Test Phase, participants were told about and played examples of the tones that now served as instructions. Before the Generalization Phase the participants were instructed to now perform the rhyme/semantic tasks. No explicit link was made in the instructions between the concrete and semantic judgments or the syllable and rhyme judgments. After the Generalization Phase participants were asked whether or not they had noticed anything else in the trials. They were then told about the primes and completed the prime-identification test.

## Results

### Prime visibility

No participant reported having noticed the primes or masks in the trials. Performance in identifying the shapes in the prime identification test at a group level was at chance (mean accuracy on forced choice task = 50.05%, SD 6.32, range 35.93–59.38, single sample comparison to 50, t(32) = 0.04, p = 0.966).

### Test and Generalization Phase accuracy

Overall, participants were very accurate in their judgments, making errors on a mean of just 0.73% (SD 0.53, range 0–2.19%) of trials in the Test Phase and on 0.55% of trials (SD 0.54 range 0–2.5%) in the Generalization Phase. Direct comparisons between error rates in the four tasks (Test Phase: concrete judgment and syllable judgment, Generalization Phase: semantic and rhyme judgment) revealed a significant overall effect of task (F(3,96) = 6.01, p = 0.001). Post-hoc analysis using Least Significant Difference (LSD) showed that errors were significantly less common on the rhyme judgment task (0.36% of trials, SD 0.46) than on all other tasks (semantic categories 0.75% SD 0.70, concrete 0.79% SD 0.61, syllable 0.67% SD 0.68; p<0.01). No other difference was statistically significant.

There was no statistically significant difference in error rates between trials requiring a different task to the previous trial (switch trials) and trials that were a continuation of the same task (non-switch trials) in the Test or Generalization Phases (Test Phase error rate on switch trials 0.65% SD 0.65, non-switch trials 0.82% SD 0.59; F(1,32) = 2.56, p = 0.12); Generalization Phase error rate on switch trials 0.54% SD 0.57; errors on non-switch trials 0.57% SD 0.60; F(1,32) = 0.11, p = 0.74). There was also no significant interaction between switch vs. non-switch trials and task type (semantic vs. phonological) in either the Test or Generalization Phases (Repeated measures ANOVA with dependent variable of percentage error and the factors of switching vs. non-switching trial type and semantic vs. phonological task for the Test Phase; switch vs. non-switch; F(1,32) = 2.52, p = 0.12, switch x task F(1,32) = 0.01, P = 0.91; for the Generalization Phase switch vs. non-switch F(1,32) = 0.156, p = 0.69, switch x task F(1,32) = 0.79, p = 0.38).

In summary, errors occurred at a low rate (<1%) across all relevant phases and trial types, the lowest being for the rhyme task. This indicates that participants understood and could do the tasks and increases the confidence we can attach to inferences drawn from the reaction time (RT) data.

### Unconscious prime – conscious instruction congruency effect: Test Phase

**Figure 2:**
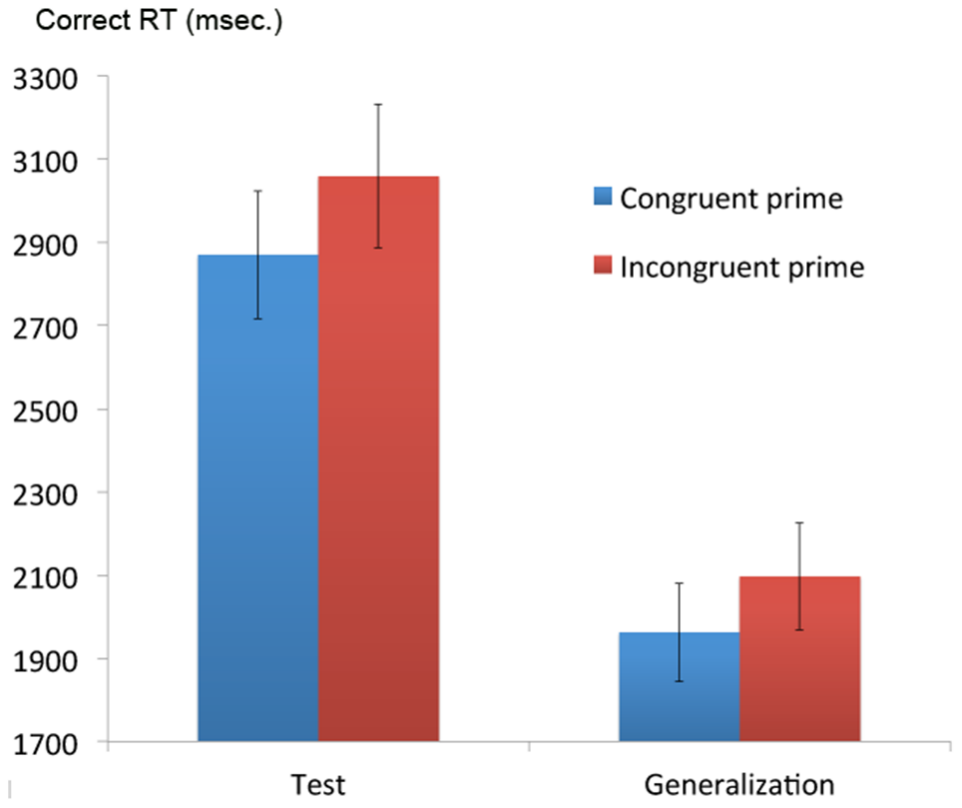
Mean correct reaction times (msec. +/− standard error) by prime-task congruency in the Test and Generalization Phases.

A repeated-measures ANOVA was conducted with the dependent variable of Test Phase correct RT and the factors of Task type (concrete vs. syllable counting), Trial Switch Status (switch vs. non-switch trial) and Congruency (prime-instruction congruent vs. incongruent). This revealed no main effect of Task type (F(1,32) = 0.50, p = 0.48); RTs were not significantly longer for concrete (mean 2777 msec. SD 849) than syllable judgment trials (2827 msec. SD 851). The main effect of Switch Status was significant with, as would be expected, RTs being significantly longer on switch compared with non-switch trials (switch trial RT = 2935 msec. SD 856; non-switch trials 2655 msec. SD 743; F(1,32) = 12.54, p = 0.001). There was no main effect of prime-task Congruency (F(1,32) = 0.13, p = 0.73) but there was a statistically significant interaction between Switch Status and Congruency (F(1,32) = 8.93, p = 0.005, effect size, partial eta squared 0.22∼Cohen's d 1.06, a ‘large effect’); on switch trials RTs were significantly faster when preceded by a congruent unconscious prime (2834 msec. SD 827) than an incongruent unconscious prime (mean 3062 msec. SD 1023; F(1,32) = 5.75, P = 0.023, see figure 2). On non-switch trials there was no statistically significant difference between congruent (2741 msec. SD 905) and incongruent primes (2563 msec. SD 690; F(1,32 = 3.29, P = 0.08) in fact a trend in the other direction. No other interactions were statistically significant.

In summary, participants demonstrated the expected switch trial RT cost and it was on switch trials that the predicted significant prime congruency effect was observed. Notably, although statistically significant overall, for 10/33 participants congruent switch trial mean RTs were very close to and sometimes even slower than incongruent trial RTs, perhaps reflecting individual differences in susceptibility to the priming effect.

### Generalization of the prime-instruction congruency effect

To examine whether the congruency effect in switching between the semantic and phonological tasks of the Test Phase would generalize, the same analyses outlined above were carried out on data from the Generalization Phase. Initially, we performed a repeated measures ANOVA on the dependent variable of correct RT with the factors of task (phonological vs. semantic), switch status (switch trials vs. non-switch trials) and prime-task congruency (congruent vs. incongruent). This revealed a significant main effect of task (F(1,32) = 82.32, P<0.001), correct responses on the semantic task were slower (2117 msec. SD 755) than on the phonological task (1631 msec. SD 648). There was also the expected significant effect of switch status with switch trials having significantly longer RTs (1986 msec. SD 816) than non-switch trials (1761 msec. SD 646; F(1,32) = 16.16, P<0.001). There was no overall effect of prime-task congruency (F(1,32) = 0.20, P = 0.661) and no statistically significant interactions (task x switch F(1,32) 1.04, P = 0.32; task x congruency F(1,32) = 0.08, P = 0.77; switch x congruency F(1,32) = 1.04, P = 0.32; task x switch x congruency (1,32) = 0.94, P = 0.34). We then compared correct RTs from only the switch trials (a mean of 34.70/64 trials were switch trials). The same general pattern was apparent as for the Test Phase; prime-instruction congruent trials showed a trend towards significant speeding (1962 msec. SD 683) relative to incongruent switch trials (2097 msec. SD 737; F(1,32) = 3.76, P = 0.061 two tailed).

However, there were good reasons to predict, a priori, that participants who had demonstrated no clear prime congruency effect during the Test Phase would be unlikely to develop it in the Generalization Phase (i.e. on a different task, and when a period – the Test Phase – had elapsed during which primes had no predictive value for task). Accordingly, to reduce the risk of a type II error, we examined whether priming generalized using data from those participants who were at or above the 50th percentile with respect to a congruency effect during the Test Phase switch trials. The mean congruency effect in the Test Phase (i.e. mean correct RT on incongruent switch trials – mean correct RT on congruent switch trials) for this group was 551 msec. (SD 318, range 168-1209) compared with -154 msec. (SD 265, range -629-155) in the remainder of the participant group.

A repeated measures ANOVA on these 16 participants' data from the Generalization Phase was then performed with the dependent variable of correct RT and the factors of task (phonological vs. semantic), trial switch status (switch vs. non-switch) and prime-task congruency (congruent vs. incongruent). This revealed that, unlike the Test Phase and as highlighted in the main Generalization Phase ANOVA above, there was a statistically significant effect of task (F(1,15) = 37.52, p<0.001) with rhyme judgments having faster RTs (1630 msec. SD 567) than semantic judgment trials (2101 msec. SD 624). There was a significant main effect of trial switch status (F(1,15) = 11.0, P = 0.005) with switch trials having longer RTs (1964 msec. SD 645) than non-switch trials (1768 msec. SD 623). There was no overall effect of congruency (F(1,15) = 0.62, p = 0.44) and no interaction between task and congruency (F(1,15) = 0.20, p = 0.67). There was, however, a statistically significant interaction between trial switch status and congruency (F(1,15) = 5.26, p = 0.037, effect size partial eta squared 0.26∼Cohen's d 1.19, a ‘large effect’); Congruent switch trials had a mean RT of 1887 msec. (SD 441), incongruent 2189 msec. (SD 670; F(1,15) = 9.67, p = 0.007). Congruent non-switch trials mean 1712 msec. (SD 520), incongruent 1671 msec. (SD 492; F(1,15 = 0.28, P = 0.60 – see figure 2).

Next, to explore whether between-subject differences in the size of the congruency effect were likely to be reliable, we examined the correlation between these effects in the Test and Generalization Phases. Simple comparison of differences in RT between the congruent and incongruent condition is likely to exaggerate any relationship as people with generally longer RTs will tend to show larger absolute differences in RTs between different conditions. Differences in RT between congruent and incongruent switch trials for each phase were therefore expressed as a proportionate change from the mean congruent RT for that phase (scaled difference  =  (RTI – RTC)/RTC, where I  =  incongruent switch trials and C  =  congruent switch trials). The magnitude of these differences in the Test and Generalization Phase were significantly correlated (Pearson's r (33) = 0.35, P<0.046). Next we sought to clarify whether the priming effect in the Generalization Phase was statistically weaker than in the Test Phase. To this end we conducted a repeated measures ANOVA on the dependent variable of correct RT with the factors of Phase (Test vs. Generalization), Task (semantic vs. phonological) Switch-status (switch vs. non-switch) and Congruency (congruent vs. incongruent primes). This revealed a statistically significant effect of Phase (F(1,32) = 98.41, P<0.001), reaction times were generally longer in the Test Phase (2800 msec. SD 1020) than in the Generalization Phase (1874 msec. SD 743), and of Task (F(1,32) 19.23, P<0.001), responses were faster overall in the phonological (2192 msec. SD 982) than the semantic (2482 msec. SD 1009) tasks. As would be expected, overall RTs on switch trials were significantly longer than on non-switch trials (F(1,32) = 23.03, P<0.001; switch 2467 msec. SD 1056, non-switch 2207 msec. SD 935). As would also be expected from the previous results, the effect of cue congruency taken across switch and non-switch trials was not statistically significant (F(1,32) = 0.22, P = 0.65). There was the expected Phase x Task interaction (F(1,32) = 6.82, P = 0.014), the differences between RT to the different phonological and semantic tasks differing between phases (see above for RT differences in the tasks). There were no interactions between Phase and Switch (F(1,32) = 0.61, P = 0.44), Task and Switch (F(1,32) = 0.50, P = 0.49), Phase, Task and Switch (F(1,32) = 0.0, P = 0.97), Phase and Congruence (F(1,32) = 0.001, P = 0.97), Task and Congruence (F(1,32) = 0.11, P = 0.75), or Phase, Task and Congruence (F(1,32) = 0.002, P = 0.97). There was however a significant Switch x Congruence interaction (F(1,32) = 7.43, P = 0.01) reflecting the greater effect of prime congruence on switch trials. There was however no statistically significant Phase x Switch x Congruence interaction (F(1,32) = 2.91, P = 0.098), the modulatory effects of switch vs. non-switch on congruence effects did not formally differ between the Test and Generalization Phases. There were no statistically significant interactions between Task, Switch and Congruence (F(1,32) = 0.05, P = 0.83) or between Phase, Task, Switch and Congruence (F(1,32) = 0.75, P = 0.39). Differences in the magnitude of priming in the Test and Generalization Phases were also examined using the RT-controlled priming effects (see above). On repeated measures ANOVA with the factor of task phase (Test vs. Generalization) the difference was not statistically significant across all 33 participants (F(1,32) = 0.0, P = 0.995) or when just the 16 who showed the greatest priming effects in the Test Phase were considered (F(1,15) = 1.13, P = 0.30), although power to detect a significant difference is of course an issue. The respective effect sizes of the priming effects in the Test and Generalization Phases were however of similar magnitude (Test 1.06, Generalization 1.19).

### Influence of prime discrimination

As discussed, at the end of the session participants were tested on their ability to discriminate the (now known) primes under the same brief, masked conditions as the earlier phases of the experiment. Whilst, on average, performance was at chance levels (see above) there was some variability. Previous research (e.g. [4], [7], [9]) suggests that the effect of cues in such task switching studies may, paradoxically, be greater when the primes are rendered less rather than more visible. To examine this issue, we first examined Pearson's correlations between individual priming effects (correct RT on incongruent trials – correct RT on congruent trials) and percentage accuracy on the prime-discrimination task. Although there was a consistency to the pattern in which the participants with the highest prime-discrimination scores tended to have the lower priming effects, neither test reached statistical significance (Test Phase priming effect-prime discrimination performance Pearson's r (33) −0.25, P = 0.17; Generalization Phase priming effect-prime discrimination r (33) −0.20, P = 0.26). Note: It was not necessary to use the RT-normalised priming effect scores described in the previous section because the current analyses did not directly contrast Test and Generalization Phases, simply the relationships between the priming effects within each and prime-discrimination performance. However, analyses using normalized scores returned almost identical results to the raw scores (Test Phase normalized priming effect-prime discrimination r(33) = −0.27, P = 0.13; Generalization Phase normalized priming effect-prime discrimination r(33) −0.26, P = 0.15).

**Figure 3:**
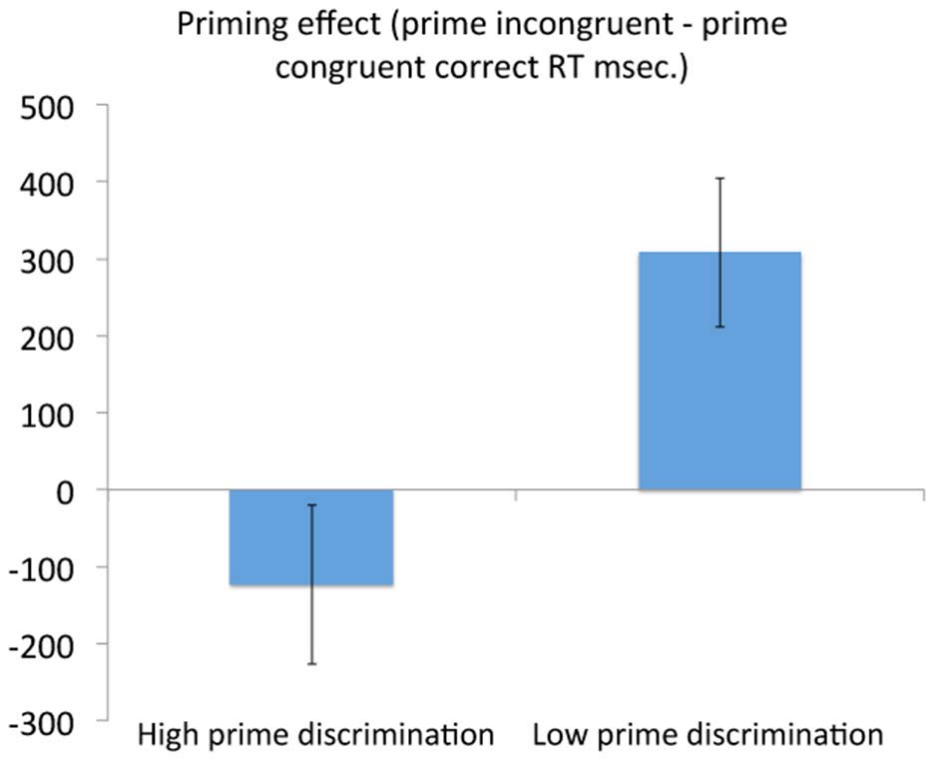
Priming effect in the Test Phase (correct RT in on prime-task incongruent trials - correct RT on prime-task congruent trials (msec. +/− standard error) for participants in the upper and lower quartile of post-test prime discrimination performance.

We then examined the extremes of the prime-discrimination continuum in a one-way ANOVA with the dependent variable of priming effect in the Test Phase (correct incongruent RT – correct congruent RT) and the factor of prime-discrimination (upper quartile vs. lower quartile). This showed that those participants scoring highest in prime-discrimination indeed had significantly lower priming effects (-124 msec. SD 401) than those with relatively low discrimination scores (308 msec. SD 374; F(1,14) = 4.95, P = 0.04 – see figure 3) despite these groups not differing in their overall RT on the Test Phase (F(1,14) = 0.042, P = 0.841). The high/low prime-discrimination effect on Generalization Phase priming effect scores was in the same direction but not statistically significant (participants with relatively high prime-discrimination scores priming effect = 52 msec.; SD 353; participants with relatively low scores 195 msec.; SD 424; F(1,14) = 0.54, P = 0.48). Participants in the upper quartile on the prime-discrimination test correctly reported the identity of the prime on 58.07% of trials (SD 1.30, range 56.25–59.38), those in the lower quartile had a mean accurate detection rate of 41.80% (SD 2.74, range 35.94–43.75).

In summary, the consistent pairing of the different primes with the phonological task of judging syllable number and the semantic task of concrete judgments in the Learning Phase exerted a significant influence over correct RTs in a subsequent Test Phase, sufficiently strongly in some participants to support a group effect despite approximately a third of participants showing little or no effect. This occurred despite the participants being unable to identify the primes even when subsequently attending to their occurrence. Interestingly, and in line with previous reports, participants who were most able to discriminate the primes at post-test showed significantly lower priming effects during the Test Phase than those least able to discriminate the primes. Participants who showed priming in the Test Phase showed a significant carry-over to the new tasks of the Generalization Phase, with susceptibility to the effect correlating in the two conditions.

## Discussion

Previous reports suggest that subliminal primes can activate ‘task sets’, mental programs that specify stimulus-response rules required to perform a specific task ([4], [7], [8], [9]). However, in all but one previous report [9], the participants had made conscious links between the symbol used as the prime and the relevant task set (i.e. the primes were identical to the subsequent consciously perceived instruction). It is possible, therefore, that the primes did little more than activate a perceptual ‘template’ that is consciously, deliberately associated with a task – rather like the ‘action triggers’ postulated by Kunde and colleagues [10].

Circumventing this limitation, Zhou and Davis [9] established prime-task associations without participants ever being consciously aware of the primes and showed that significant congruency effects nevertheless occurred. Using identical training/test phases, albeit with a modified task and different equipment, we have replicated this extraordinary finding here; it appears possible to subliminally prime ostensibly volitional changes in task-set.

The key finding from the current study concerns the level at which such priming can occur. We have shown, for the first time, that priming by cues that lie outside of conscious awareness can extend beyond the specific trained task to novel tasks that require related processes. That primes linked with a syllabic enumeration task influenced subsequent rhyme judgments strongly suggests that a switch to more general process of phonological analysis (as well as, potentially, activity specific to syllable counting) was being facilitated. Similarly, that a subliminal cue linked with judging concreteness influenced subsequent judgments of whether “defense” and “guilty” were related strongly indicates that a switch to a ‘semantic mode’ (in addition to activity specific to concrete/abstract distinctions) was primed.

Although formal testing detected no statistically significant difference in priming magnitude between Test and Generalization, and the effect sizes were of a similar magnitude, there are reasons to believe that the effects could tend to be weaker. In addition to having different tasks, the Generalization phase was separated from the Training phase by a long interval (the Test Phase) in which primes no longer reliably predicted task. An interesting manipulation for future studies would be to repeat the 100% prime-task predictive Training phase before switching to the generalization tasks to estimate the specific effects of task-change on priming.

Some previous studies [4], [9] have reported unconscious priming-congruency effects based on all trials in the task and not delineated effects for switch and non-switch trials. As here, Reuss et al. (study 2) reported effects of masked primes only on switch trials (in their case only on accuracy)[8]. In our study, non-switch trials in the Test phase showed a trend for faster responses following incongruent primes, a potentially interesting effect for which we have no explanation. However, that this was absent in the Generalization phase suggests that it may not be reliable. There are grounds to expect that switch and non-switch trials may differ. Faster RTs on non-switch trials indicate that the correct task set was already in place from the previous trial(s). An unconscious prime that is congruent with both the preceding trial and the subsequent conscious instruction for the next trial (i.e. on a non-switch trial) may have little detectable additional impact in facilitating maintenance of the appropriate task set. On switch trials, by definition, there can be no such useful carry-over of task, hence the influence of the primes may be more easily detected. Further work is required to establish the reliability of this switch/non-switch trial difference.

Our results suggest that individual differences in how strongly the congruency effect appeared in the Test Phase were broadly reflected in the Generalization phase. It would be surprising if this were not the case. We cannot infer from this correlation that there are individual differences in susceptibility to unconscious task-set priming that are reliable across tasks as we had only one training phase in the study.

About a third of the participants showed little evidence of congruency effects in the Test phase, with the remainder showing it sufficiently strongly to return the overall group result. Various factors have been linked to priming susceptibility including perceptual and inhibitory differences but it is unclear how much these generalize over tasks and contexts [11], [12]. Two previous reports [4], [9]have suggested that priming in similar task-switching paradigms may be weakened when the primes are made more visible. Whilst we did not manipulate visibility the finding of significantly lower priming effects in participants in the top quartile on the prime-discrimination test (in the absence of any general RT differences) was consistent with this suggestion. It has been argued in other priming contexts that masked unconscious primes are not processed as separate perceptual events but contribute to the overall accumulation of evidence leading to a response [13], [14]. In the Test (and Generalization) phases in the current study, the primes are not useful to task performance because they are as likely to be incongruent with the subsequent instruction as congruent. The result would therefore be consistent with greater awareness of the prime as a separate perceptual event allowing participants to discount their influence in a way that is less possible for participants who, by inference from the prime-discrimination test, are less aware of their occurrence; paradoxically it may be harder to ‘ignore’ something of which you are not consciously aware.

The idea that events that lie outside of conscious awareness may influence cognitive function and behavior is already well established. It would be surprising in many ways if they did not. Some contemporary accounts view the brain as trying to build optimal statistical models of events from imperfect data to guide action (e.g. [15]). It would make little sense for entry to those models was limited to the subset of information to which we have conscious access. In the tasks used in this study, as almost invariably occurs across any range of measures, participants become progressively faster in making their responses. This can be related to repeated experience of making the kind of decisions required but also growing familiarity with the task structure and the repetition of intervals, locations etc. within trials. These can guide the orchestration of attention and mental content to contribute to progressively faster responses without participants necessarily being conscious of this process. We have demonstrated that subliminal priming of task-switching can generalize to a new task, but here the novel task was embedded in an identical trial structure to that in which the priming was first acquired. An interesting question for the likely ecological significance of the effect is whether such generalization is robust to temporal and spatial variations or is limited the invariant structure of our trials.

In summary the results of this study were consistent with previous reports in showing that reliable predictive relationships between two brief, masked visual primes and two task-sets during a Training phase resulted in significant prime-task congruency RT effects in a subsequent Test phase, despite participants being, on average, entirely unable to discriminate the prime shapes on post-test. The current study extended previous findings in showing that the prime influence generalized to a novel task, consistent with priming occurring at the level of process as well as, or rather than, the highly specific task. Although the current study did not manipulate prime visibility, interestingly, individual differences in participants' ability to discriminate the primes at post-test was, at the extremes of the continuum, related to individual variability in the magnitude of priming during the Test phase.
